# Topical Delivery of Coenzyme Q10-Loaded Microemulsion for Skin Regeneration

**DOI:** 10.3390/pharmaceutics12040332

**Published:** 2020-04-07

**Authors:** Kyeong-A Ryu, Phil June Park, Seong-Bo Kim, Bum-Ho Bin, Dong-Jin Jang, Sung Tae Kim

**Affiliations:** 1 Institute of Digital Anti-Aging Healthcare, Inje University, Gimhae 50834, Korea; rkakk95050@oasis.inje.ac.kr; 2 Department of Genetic Engineering, Sungkyunkwan University, Suwon-si, Gyeonggi-do 16499, Korea; philjunep@gmail.com; 3 Bio-Living Engineering Major, Global Leaders College, Yonsei University, 50 yonsei-ro, Shinchon-dong, Seodaemun-gu, Seoul 03722, Korea; seongbo.kim@yonsei.ac.kr; 4 Department of Biological Sciences, Ajou University, 206 Worldcup-ro, Yeongtong-gu, Suwon 16499, Korea; bhb@ajou.ac.kr; 5 Department of Pharmaceutical Engineering, Inje University, Gimhae 50834, Korea

**Keywords:** coenzyme Q10, microemulsion, topical administration, skin regeneration

## Abstract

The aim of this study was to develop a coenzyme Q10 (CoQ10) microemulsion system with improved solubility, penetration, and wound healing efficacy. Based on the pseudo-ternary diagram, microemulsions containing isopropyl myristate (IPM), Cremophor EL^®^, and Transcutol^®^ HP were selected and confirmed to be nanosized (<20 nm) and thermodynamically stable based on the dilution and thermodynamic stability tests. The CoQ10-loaded microemulsion with a surfactant/co-surfactant (S/CoS) ratio of 2:1 (*w*/*w* %) demonstrated a higher permeation efficacy compared to microemulsions with S/CoS ratio of 3:1 or 4:1 (*w*/*w* %). Additionally, the CoQ10-loaded microemulsion with an S/CoS ratio of 2:1 demonstrated a relatively rapid wound healing effect in keratinocytes and fibroblasts. Overall, these data suggest that a microemulsion based on IPM, Cremophor EL^®^, and Transcutol^®^ HP could be an effective vehicle for the topical administration of CoQ10 and could be utilized for the application of other therapeutic agents that have difficulty in penetrating the skin.

## 1. Introduction

Skin, the outermost layer of the body, is the first protective barrier against external physical (e.g., ultraviolet and moisture), chemical (e.g., toxin and drug), and biological factors (e.g., allergen and pathogen) [1,2,3]. The protective function of the skin is mainly due to the stratum corneum, which belongs to the epidermis, a stratified squamous epithelium [4,5]. The stratum corneum is the superficial layer of the epidermis, directly in contact with external stimuli [6]. The stratum corneum consists of differentiated corneocytes and hydrophobic lipids, often described as having a “brick and mortar” type of structure in contrast to other tissues in the body [7]. On average, approximately 20 layered corneocytes embedded in the lipids prevent the loss of water, as well as block the entry of unwanted substances including drugs which is useful in protecting our body and maintaining homeostasis [8].

The skin is a potential route for the administration for local and systemic therapeutics with several advantages including the avoidance of first-pass metabolism in the liver, ease of self-administration, and increased patient compliance [9]. In the case of skin ailments, transdermal drug delivery is practically effective as direct application allows higher drug accumulation at the target region with minimal side effects (e.g., systemic toxicity) compared to oral drug delivery [10]. However, due to the skin barrier, the penetration of therapeutics across the stratum corneum remains challenging. Especially, general therapeutic agents demonstrate a considerably low penetration efficacy unlike moderately lipophilic substances (molecular weight < 500 Da and log *P* of 1 to 4) [11]. Hence, these therapeutic agents require skin penetration enhancement strategies such as physical permeation enhancement (e.g., iontophoresis and microneedle) [12], chemical permeation enhancement (e.g., chemical enhancer and ionic liquid) [13], and nanocarrier-based permeation enhancement (e.g., microemulsion and transfersomes) [14,15].

Microemulsions are thermodynamically stable vehicles that contain oil and water stabilized by amphiphiles such as surfactants and co-surfactants [16]. Such microemulsions are transparent, isotropic colloidal systems with a droplet size less than 100 nm and have been widely studied since microemulsions were defined [17,18]. As previously reported, compared with conventional emulsions, these systems demonstrate enhanced transdermal permeation owing to their smaller size and disruption of the stratum corneum [19,20]. In addition, these systems are conventionally advantageous due to the fact of their pharmaceutical stability, easy fabrication, and scale-up [21]. Therefore, microemulsions are preferred in dermatological cosmetic applications over the past decades.

Coenzyme Q10 (CoQ10, Mw = 863.3 g/mol) is a quinone with side chains of 10 isoprenoids [22]. CoQ10 generally plays an important role as an electron carrier in the oxidative phosphorylation process of the mitochondria while acting as an antioxidant outside the mitochondria [23]. Recently, CoQ10 has gained attention for its therapeutic application for several disorders including cardiovascular diseases, diabetes mellitus, and even cancer [24]. For example, previous literature has reported that CoQ10 intake reduces the progression of chronic heart failure, angina pectoris, arrhythmias, and ventricular dysfunction [25,26,27]. In addition, other reports have indicated that CoQ10 demonstrated anti-inflammatory and wound healing properties [28,29]. However, due to the fact of its poor water solubility and relatively high molecular weight, CoQ10 is unsuitable for skin delivery despite its beneficial effects. To overcome its low skin permeation, effective and noninvasive microemulsion carriers are essential owing to their low potential for toxicity and skin irritation.

The aim of this study was to develop a novel CoQ10-loaded microemulsion system to increase skin delivery and skin regeneration. To confirm this, the physicochemical properties of the microemulsion were evaluated to better understand the topical application of CoQ10. In addition, the cell scratch assay was performed to confirm wound healing efficacy.

## 2. Materials and Methods

### 2.1. Materials

CoQ10, isopropyl myristate (IPM), and oleic acid (OA) were purchased from Tokyo Chemical Industry (TCI, Tokyo, Japan). Labrafil M1944, Labrasol, and Transcutol^®^ HP were provided by Gattefosse (Gattefosse, France). Cottonseed oil was obtained from Daejung Chemicals (Daejung, Korea). Tween 80, propylene glycol (PG), and polyethylene glycol (PEG) 400 were procured from the Duksan Corporation (Duksan, Korea). Cremophor EL^®^ (Kolliphor) was purchased from Sigma–Aldrich (St. Louis, MO, USA). Ethyl alcohol and isopropyl alcohol were of HPLC grade (Duksan, Korea). For the in vitro cell experiments, fetal bovine serum (Gibco^®^, Grand Island, NY, USA), bovine calf serum (Gibco^®^, Penrose, AKL, NZ), penicillin–streptomycin (Corning^®^, Manassas, VA, USA), 0.05% trypsin 0.53 mM EDTA (Corning^®^, Manassas, VA, USA), and Dulbecco’s modified Eagle’s medium (Corning^®^, Manassas, VA, USA) were used. For the cell proliferation test, a cell counting kit (CCK-8) was used (Dojindo Molecular Technologies, Inc., Kumamoto, Japan). All other purchased reagents were of analytical grade.

### 2.2. Selection of Oils, Surfactants, and Co-Surfactant Mixtures for Microemulsions

Prior to microemulsion formulation, the solubility of CoQ10 was screened in various oils, surfactants, and co-surfactants. The oils selected were IPM, OA, Labrafil M1944, cottonseed oil, and a mixture of IPM and OA in a ratio of 2:1 and 3:1 (*w*/*w* %). An excess amount of CoQ10 was added to 500 μL of each oil. Then, each sample was vortex-mixed and maintained using an isothermal shaker at body temperature (37 °C) for 24 h to reach an equilibrium state. After centrifugation at 2000 rpm for 10 min, each supernatant was harvested and quantitatively determined using high-performance liquid chromatography (HPLC) (Hitachi LaChrom Elite^®^, Tokyo, Japan).

### 2.3. Quantitative Analysis of CoQ10

Each supernatant was diluted using a mobile phase consisting of a mixture of ethyl alcohol–isopropanol (75:25 *v*/*v* %). Then, the C18 column (4.6 × 150 nm, i.e., 5 μm particles, YoungJin BioChrom Aegispak, Seongnam-si, Korea) was used for separation. All experiments were performed using the HPLC L-2000 series (Hitachi LaChrom Elite^®^, Tokyo, Japan) equipped with diode array detector, an autosampler (L-2200), a column oven (L-2300), and a pump (L-2130) under the following condition: injection volume (20 μL), the flow rate (1 mL/min), detection (275 nm), and column temperature (37 °C).

### 2.4. Construction of Pseudo-Ternary Phase Diagram

The pseudo-ternary phase diagrams of the oil selected, water, and the mixture of surfactant and co-surfactant were constructed by the titration method at room temperature. Optimum hydrophilic and lipophilic balance (HLB) levels of the experimental surfactant and co-surfactants ranged between 10 and 13, which is available for preparing the o/w microemulsion. Prior to experimental approaches, the surfactants theoretically selected were Labrasol, Tween 80, and Cremophor EL^®^. In addition, the co-surfactants used were Transcutol^®^ HP, PG, and PEG 400, which are widely used as pharmaceutical ingredients.

First, the experimental study selected an appropriate surfactant. In brief, 5 μL of each type of oil was added into 3 mL of each surfactant solution (15 *w*/*v* %) using a pipette (PIPETMAN^®^, Gilson, France) under mild magnetic stirring until slightly cloudy. During titration, each sample was stirred to reach an equilibrium state and the quantity of oil needed for emulsification was recorded. Furthermore, to evaluate the emulsification ability of the surfactants described above, the oil and surfactant mixtures were homogenized and then diluted with double-distilled water. The prepared emulsions were kept for 1 h and the transmittance was assessed at 650 nm using a UV spectrometry. An appropriate co-surfactant was also selected in the range of the microemulsion region formed by the oil and surfactant. First, the surfactant/co-surfactant (S/CoS) mixture was fixed at a ratio of 1:1 and the pseudo-ternary diagrams were constructed based on the following weight ratios of oil and S/CoS mixtures: 1:9, 1:8. 1:7, 1:6, 1:5, 1:4, 1:3, 1:2, 1:1, 1:0.7, 1:0.43, and 1:0.11.

The pseudo-ternary phase diagrams were constructed using the water titration method. In brief, double-distilled water was added dropwise to a certain amount of oil and the S/CoS mixture selected and agitated at room temperature. During titration, the boundaries between the microemulsion and other phases were monitored when the solutions appeared cloudy, and were evaluated in terms of turbidities and transparency.

### 2.5. Preparation of CoQ10-Loaded o/w Microemulsion

Based on the preliminary experiment using phase diagrams, various formulations with CoQ10 were prepared using oil ranging from 5% to 15%, S/CoS mixture ranging from 30% to 60%, and double-distilled water ranging from 40% to 60%. In brief, CoQ10 was gently dissolved into the pre-weighed mixture of oil and S/CoS. The indicated amount of water was added and sonicated until a transparent liquid was obtained. In order to optimize the CoQ10-loaded o/w microemulsion, all formulations were evaluated using the dilution test as well as the thermodynamic stability test.

### 2.6. Characterization of CoQ10-Loaded o/w Microemulsion

#### 2.6.1. Stability Assessment 

Briefly, for the dilution test, 500 mg of each formulation was diluted with 50 mL of double-distilled water. Twenty-four kinds of microemulsions were defined as clear when the mixture appeared optically transparent or slightly turbid when the mixture appeared optically cloudy.

The selected formulations were subjected to the thermodynamic stability tests, including the centrifugation test, heating–cooling cycle, and freeze–thaw test, after the dilution test. In each test, the presence of phase separation, creaming or cracking of microemulsion was checked, and the following experiment was carried out. For the centrifugation test, an accelerated stability test which is based on the basic principle that two-phase regimes with different densities (e.g., oil and water) separate due to centrifugal force, each formulation was first centrifuged down at 3000 rpm for 30 min. Then, phase separation was observed. For the heating–cooling cycle test, each formulation was examined at room temperature for 48 h after 6 cycles of heating (45 °C) and cooling (4 °C). For the freeze–thaw cycle test, each formulation was observed 48 h after 3 cycles of freezing (−21 °C) and thawing (25 °C) experiments; during these experiment, drug content were not determined.

#### 2.6.2. Entrapment Efficacy of CoQ10 in o/w Microemulsion

The total amount of CoQ10 was measured using the HPLC method, as described above. after completely dissolving the microemulsions by ethyl alcohol–isopropanol mixture (75:25 *v*/*v* %). The percentage of drug content entrapped in the microemulsion was estimated as the following equation: % drug content = (actual amount of CoQ10/theoretical amount of CoQ10) × 100 (%).

#### 2.6.3. Measurement of Droplet Size and Zeta Potential Value

The mean diameter and zeta potential of the microemulsion were measured using a Malvern ZetaSizer (NanoZS90, Malvern instrument, Malvern, UK) at 25 °C with a fixed angle of 90°. The data were represented in triplicate. The zeta potential was also measured and represented in triplicate.

### 2.7. In Vitro Cell Experiment

#### 2.7.1. Preparation of Cells

The HaCaT cells were cultured in Dulbecco’s modified Eagle’s medium, supplemented with 10% fetal bovine serum (Gibco^®^, Grand Island, NY, USA) and 1% penicillin–streptomycin solution (Corning^®^, Manassas, VA, USA). The NIH3T3 fibroblast cells were cultured in Dulbecco’s modified Eagle’s medium supplemented with 10% bovine calf serum (Gibco^®^, Penrose, AKL, NZ) and 1% penicillin–streptomycin solution (Corning^®^, Manassas, VA, USA). Both cells were cultured in a humidified 5% CO_2_ atmosphere at 37 °C and prepared for the cell proliferation assay and wound healing scratch assay.

#### 2.7.2. Cell Proliferation Assay

The cell proliferation assay was performed using a colorimetric assay, the CCK-8 kit (Dojindo Molecular Technologies, Inc., Kumamoto, Japan). In brief, both cell lines (1 × 10^5^ cells/well) were preliminarily seeded in 24 well plates and cultured in a humidified 5% CO_2_ atmosphere at 37 °C. After overnight incubation, the cell culture medium was replaced by the CoQ10-loaded microemulsion containing medium ranging from 0.5 to 2 mg/mL. After a 4 h inoculation, the CCK-8 solution was added to each well as 10% of the total volume of the medium and additionally incubated for 4 h in the incubator. The absorbance was measured at 450 nm using a microplate reader (SYNERGY HTX, BioTek, Winooski, VT, USA). Based on data, cell proliferation was calculated according to the following formula: cell proliferation (%) = ((A_sample_ − A_b_)/(A_c_ − A_b_)) × 100 (%), (in this formula, A_sample_ = absorbance in CoQ10-loaded microemulsion-treated group, A_b_ = absorbance in blank, and A_c_ = absorbance in control group).

#### 2.7.3. Wound Healing Scratch Assay

The HaCaT and NIH3T3 cells were seeded into a Scar^TM^ Block (Cat. No. 201935, SPL, Pocheon-si, Korea) to reduce the cell debris formed during cell scratching and cultured as a monolayer with high confluence after cell seeding (1 × 10^4^ cell/25.5 mm^2^). Prior to the experiment, the frame of the block was gently removed using sterile tweezers to allow uniform scratching at the same intervals. Then, the cells were washed with phosphate-buffered saline to remove floating cells or debris. After cell preparation as a monolayer, the CoQ10-loaded microemulsion (1 mg/mL) was inoculated with the fresh culture medium. Cell migration was observed at 0, 18, 24, and 36 h after treatment with the microemulsion and images were obtained using a phase-contrast microscope (KI-400, Korea Lab Tech, Korea). The wound area was calculated by tracing the cell-free zone in each image using the Image J software (ImageJ 1.52a, Wayne Rasband, Bethesda, MD, USA). Each wound area was reduced to a function of time and was explained as the percent wound healing area for a better understanding of cell migration as follows: wound healing area % = (A*_t=0h_* – A*_t=__Δh_*)/A*_t=0h_* × 100 (%), where A*_t=0h_*is the wound area at the initial time, and A*_t=__Δh_* is the wound area measured at each time point after scratching [30].

### 2.8. Permeation Experiment

#### 2.8.1. In Vitro Membrane Permeation Experiment

A semipermeable polycarbonate membrane (0.4 μm, Whatman^TM^ 800282 Nuclepore^TM^, GE Healthcare Bio-Sciences Corp., NJ, USA) was placed between the donor compartment and the receptor compartment of the Franz diffusion cell apparatus. Then, 9 mL of medium was filled in the receptor compartment without a bubble beneath the membrane at 37 °C. After preparation, the CoQ10-loaded o/w microemulsion was loaded into the donor compartment. Two grams of each microemulsion were loaded in the donor compartment. Simultaneously, the same amount of CoQ10 dispersed in 1% sodium lauryl sulfate solution was loaded in the control. The CoQ10 penetration was monitored and quantitatively analyzed by HPLC as described above.

#### 2.8.2. In Vitro Skin Permeation Experiment

The skin permeation study was performed using a Franz diffusion cell apparatus with an effective penetration area (1.43 cm^2^) and a receptor compartment. Freshly excised skin of female hairless mice (SKH-1) were harvested and used after approval of the Institutional Animal Care and Use Committee (IACUC) protocol (IACUC-Inje University 2019-015) (approval date: 26 September 2019). In brief, murine skin was prepared after removing the adherent fat layer and washed with phosphate-buffered saline (pH 7.4). Then, the skin was placed between the donor and receptor compartments of the Franz diffusion cell apparatus system. Next, 9 mL of medium was filled in the receptor compartment without a bubble beneath the skin. Likewise, after preparation of the system, 2 g of each microemulsion selected was loaded in the donor compartment. In addition, the same amount of CoQ10, dispersed in 1% sodium lauryl sulfate solution, was loaded in the control. One day after treatment, the skin was harvested and rinsed with phosphate-buffered saline to remove the excess CoQ10 on the skin. Each skin was sliced and sonicated for extracting the CoQ10 inside the skin.

### 2.9. Statistics

The student *t*-test and analysis of variance (ANOVA) were performed to compare the difference of mean values among samples. When *p*-value was less than 0.05, the result was considered significant: *, *p* < 0.05; **, *p* < 0.01; and ***, *p* < 0.001.

## 3. Results

### 3.1. Screening of CoQ10 in Oils, Surfactants, and Co-Surfactants

The solubility of CoQ10 was screened in various oils, surfactants, and co-surfactants. As shown in Table 1, CoQ10 demonstrated different levels of solubility depending on the type of oil and mixtures. As a result, the solubility of CoQ10 was sequenced in the order of IPM > IPM:OA (3:1) > IPM:OA (2:1) > cottonseed oil > oleic acid > Labrafil M1944. The IPM was selected as the oil phase for the CoQ10-loaded microemulsion due to the highest solubility (209.88 ± 7.42 mg/mL) in the given experiment.

**Figure 1:**
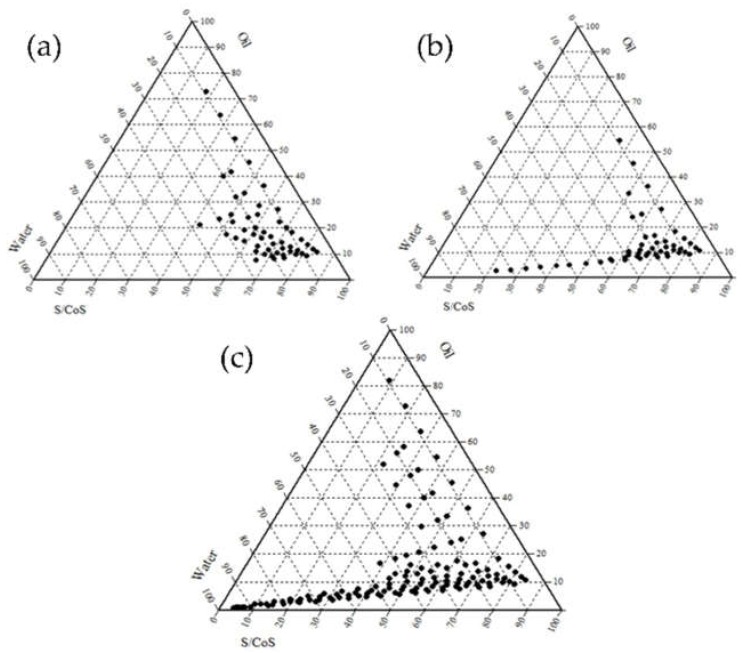
Pseudo-ternary phase diagram of microemulsion composed of oil (IPM), surfactant (Labrasol, Tween 80 and Cremophor EL)-co-surfactant (Transcutol HP^®^) mixture (1:1 ratio, *w*/*w*), and water (**a**) oil (IPM), S/CoS (Labrasol:Transcutol^®^ HP, 1:1); (**b**) oil (IPM), S/CoS (Tween 80:Transcutol^®^ HP, 1:1); (**c**) oil (IPM), S/CoS (Cremophor EL: Transcutol^®^ HP, 1:1).

**Figure 2:**
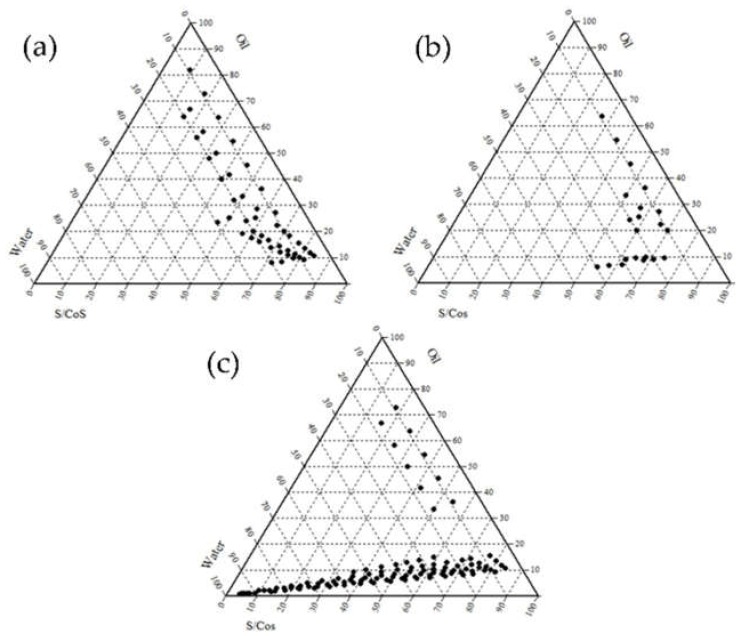
Pseudo-ternary phase diagram of microemulsion composed of oil (IPM), surfactant (Labrasol, Tween 80 and Cremophor EL^®^)-co-surfactant (PG) mixture (1:1 ratio, *w*/*w*) and water (**a**) oil (IPM), S/CoS (Labrasol:PG, 1:1); (**b**) oil (IPM), S/CoS (Tween 80:PG, 1:1); (**c**) oil (IPM), S/CoS (Cremophor EL^®^: PG, 1:1).

Three types of nonionic surfactants with a high HLB value (>10) were evaluated due to the fact of their low toxicity and irritation. Prior to the experimental approach, Cremophor EL^®^, Labrasol, and Tween 80 were selected for optimum S/CoS, presumably resulting in a stable o/w microemulsion. To investigate the solubility of CoQ10 in each substance, experimental approaches were performed as presented in Table 1. The solubilizing capacity of the surfactant for IPM was sequenced in the order of Labrasol > Cremophor EL^®^ > Tween 80. Additionally, the emulsification ability was sequenced in the order of Tween 80 > Cremophor EL > Labrasol. Based on these studies, to determine an appropriate S/CoS mixture, the microemulsion regions are illustrated as shown in Figure 1 and Figure 2. Figure 1 illustrates pseudo-ternary phase diagrams of the microemulsion, composed of IPM with the surfactant (Labrasol, Tween 80, or Cremophor EL^®^) and Transcutol^®^ HP as the co-surfactant, with a constant S/CoS ratio of 1:1. As a result, the Cremophor EL^®^:Transcutol^®^ HP mixture (Figure 1c) demonstrated a relatively wider range of microemulsion than the mixture of Labrasol:Transcutol^®^ HP mixture (Figure 1a) as well as Tween 80:Transcutol^®^ HP mixture (Figure 1b). Figure 2 presents the pseudo-ternary diagram composed of IPM, surfactant, and PG as the co-surfactant, with a constant S/CoS ratio of 1:1. As a result, the Cremophor EL^®^:PG mixture (Figure 2c) also demonstrated a wider range of microemulsion than other mixtures (Figure 2a,b). Compared to PG, the use of Transcutol^®^ HP provided a relatively wider range of microemulsion in Figure 1 and Figure 2. Collectively, our findings demonstrated that Cremophor EL^®^ and Transcutol^®^ HP were suitable for a microemulsion composed of IPM in the given experiments.

### 3.2. Phase Behavior

**Figure 3:**
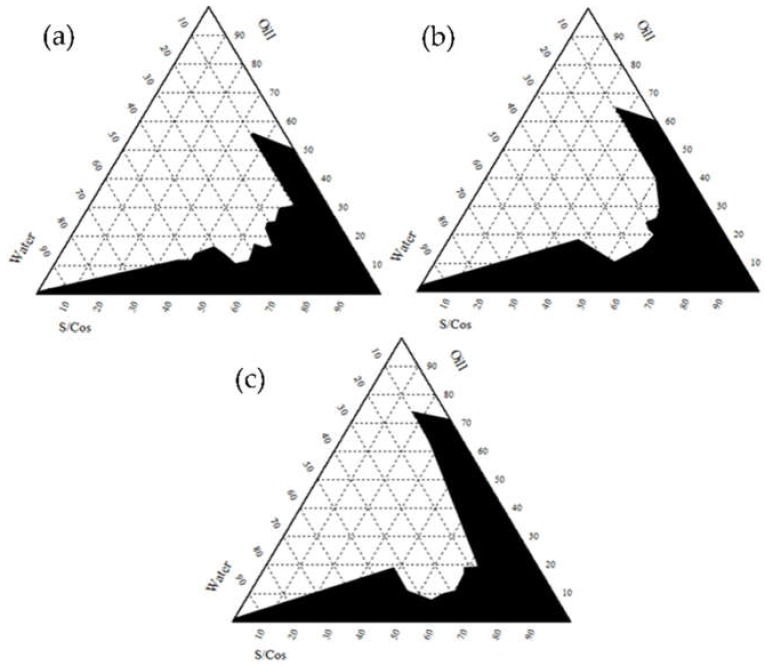
Pseudo-ternary phase diagram of oil (IPM), surfactant (Cremophor EL^®^)-co-surfactant (Transcutol^®^ HP) and water (**a**) Cremophor EL^®^:Transcutol^®^ HP (2:1, *w*/*w*); (**b**) Cremophor EL^®^:Transcutol^®^ HP (3:1, *w*/*w*); and (**c**) Cremophor EL^®^:Transcutol^®^ HP (4:1, *w*/*w*).

To construct an optimized microemulsion, the pseudo-ternary phase diagrams were illustrated for investigating the existence of microemulsion regions. Figure 3 demonstrates the phase behaviors of IPM (oil), Cremophor EL^®^ (surfactant), and Transcutol^®^ HP (co-surfactant), where the ratios of the Cremophor EL^®^:Transcutol^®^ HP mixture were 2:1, 3:1, and 4:1 (*w*/*w* %), respectively. Hence, microemulsions were fabricated under specific conditions, indicated as dark areas in each diagram. In brief, the formulation was judged as “good” when a transparent microemulsion was formed with good flowability; the formulation was judged as “poor” when the formulation demonstrated turbid behavior or no formulation was observed after the end of the experiment. In addition, the microemulsion was judged as “poor” when the mixed formulation became highly viscous like a gel, indicating no changes in the meniscus after being tilted to a 90° angle. Overall, it was considered “good” in the regions forming microemulsions, indicated as dark areas. Based on such criteria, the pseudo-ternary phase diagrams were constructed to identify and optimize the microemulsion suitable for CoQ10. In all diagrams, the microemulsion tended to be formed as the S/CoS increased. An increased S/CoS ratio provided the environment suitable for micelle formation, as well as the solubilizing capacity in the system.

### 3.3. Optimization of CoQ10-Loaded o/w Microemulsion

Among 864 formulations, 24 formulations (F1 to F24) were selected and prepared to separate the efficient microemulsion formulation with a high drug entrapment and thermodynamic stability in a given experiment as shown in Table 2. Based on the dilution test, the stabilities of emulsions were indicated as follows: the microemulsion was judged as “clear” when the prepared emulsion was transparent, whereas it was judged as “turbid” when precipitation was formed or phase separation occurred. Among the 24 formulations, four formulations (F21 to 24) appeared turbid upon dilution with water at ratio 1:100 *v*/*v* % and demonstrated a poor thermodynamic stability. The remaining formulations showed transparent behavior resulting from the dilution test. Based on the results of the thermodynamic stability test, six formulations (F1, F2, F9, F10, F17, and F18) containing 0.5 or 1% CoQ10 (*w*/*w* %), were thermodynamically stable following tests of centrifugation, heating-cooling, and freeze–thaw test. Therefore, among the six formulations, the following formulations (F2, F10, and F18) were selected when considering the drug content; for example, F2 was selected because the amount of CoQ10 was two-fold higher than that of F1. Hence, in the given experiments, the stable formulations containing 1% CoQ10 (*w*/*w* %) were F2, F10, and F18.

### 3.4. Physicochemical characterization of CoQ10-loaded o/w microemulsion

Three formulations (i.e., F2, F10 and F18) were selected and optimized for optimal composition after a dilution test and thermodynamic tests. Each droplet size and zeta potential of the CoQ10-loaded microemulsions is presented in Table 3. In addition, the drug content of CoQ10 and pH of formulations were measured. The results demonstrated that the size of each microemulsion was less than 20 nm with no significant difference. Although the polydispersity index of F2 was relatively low compared to that of F10 and F18, all formulations demonstrated uniform droplet size due to the low polydispersity index close to 0. The zeta potential value of each formulation was slightly negative, which was ranged from −11 to −15 mV. In addition, the drug content of each formulation was approximately 100%, implying that CoQ10 was well-entrapped in the oil phase consisting of IPM, regardless of the S/CoS ratio. Collectively, F2, F10, and F18 demonstrated no significant differences in terms of physicochemical properties of the microemulsion, although they had different compositions and S/CoS ratios so far.

### 3.5. Permeation Experiments

**Figure 4:**
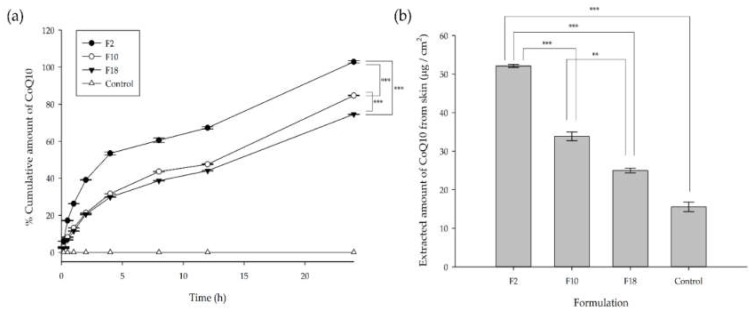
Cumulative percent release of CoQ10 as a function of time and accumulation of CoQ10 (**a**) release of CoQ10 from F2, F10, and F18 in the Franz diffusion cell using a semipermeable membrane; (**b**) extracted amount of CoQ10 from the skin after 24 h in the Franz diffusion cell using a murine skin. Data are presented as mean ± SD (*n* = 3); ** *p* < 0.01; *** *p* < 0.001.

Figure 4 showed the cumulative release of CoQ10 entrapped in microemulsions including F2, F10, and F18 in the dissolution media for 24 h in vitro as shown in Figure 4a. Compared to other formulations, F2 demonstrated a higher permeation profile of CoQ10 in the Franz diffusion cell apparatus using a semipermeable membrane. Additionally, 8 h after the initial release, F10 showed a statistically higher permeation profile than F18. Comparing F10 and F18, F2 demonstrated a lower S/CoS ratio, improving the release of CoQ10 presumably due to the S/CoS synergism on the CoQ10 solubilization capacity and permeation characteristics. In parallel to the permeation test, the additional release study using the dialysis membrane further demonstrated that F2 had a higher CoQ10 release profile than other formulations as shown in Figure S1.

Prior to the skin penetration experiment, various o/w microemulsions were prepared. The selected microemulsions containing 1% CoQ10 (F2, F10 and F18) were prepared based on the constructed phase diagram and compositions as shown in Table 2. As shown in Figure 4a, F2, F10, and F18 did not demonstrate an initial burst release; particularly, F2 demonstrated a higher permeation profile than other formulations. Such profiles correlated to the accumulation of CoQ10 through the stratum corneum in the skin as shown in Figure 4b. The amount of CoQ10 in the skin (F2) was 1.54 fold higher than that of F10 and 1.77 fold higher than that of F18. Furthermore, the amount of CoQ10 in the skin was 3.35 fold higher than that of the control. Hence, the microemulsions indicated effective skin permeation. F2 showed the highest CoQ10 accumulation in the skin compared to F10 and F18 (Figure 4b) which was well-matched with the in vitro data (Figure 4a).

### 3.6. Effect of CoQ10 on Skin Regeneration

**Figure 5:**
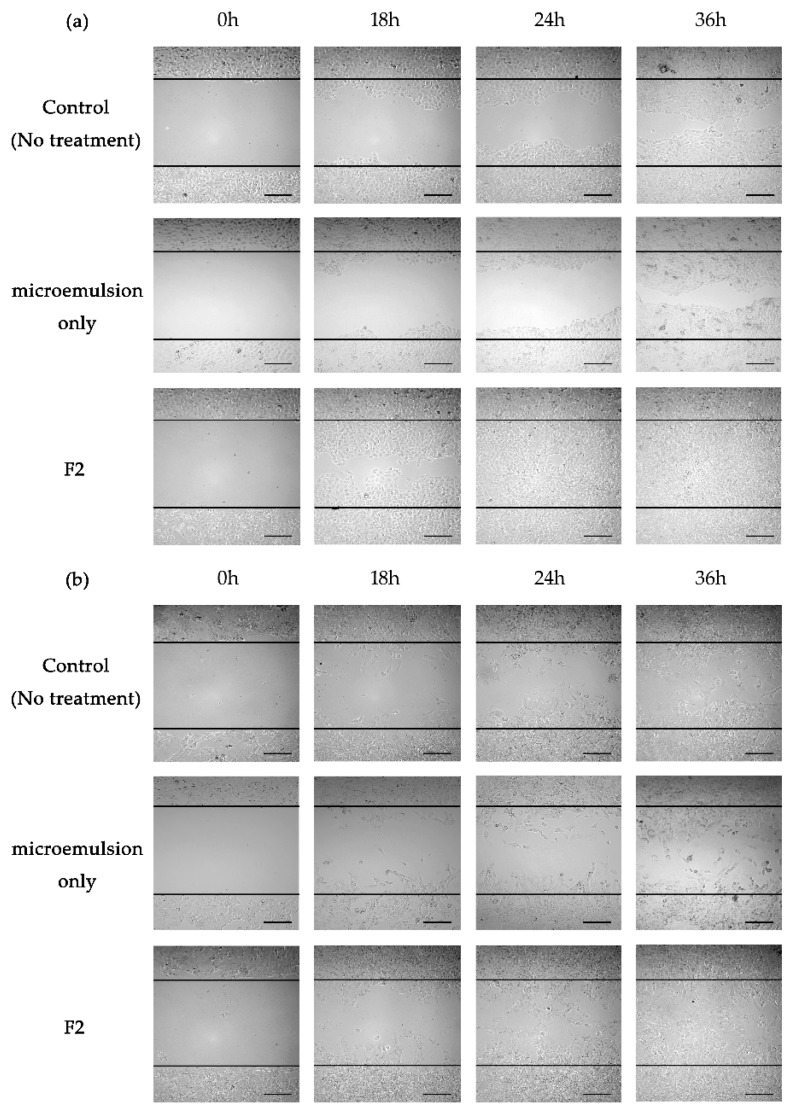
In vitro scratch assay for skin regeneration (**a**) HaCaT keratinocyte cell line; (**b**) NIH3T3 fibroblast cells were scratched and treated with or without F2 containing CoQ10 (1 mg/mL). Each image was taken at 0, 18, 24, and 36 h after the treatment (Scale bar, 200 μm).

Prior to the skin regeneration study, cell proliferation was first assessed in HaCaT keratinocyte cells and NIH3T3 fibroblast cells, the representative skin cell lines (Figure S2). Compared to the control, F2 demonstrated no cytotoxicity and even improved the proliferation of both cell lines in each set of experiments. Based on the data, the cell scratch assay, also known as the wound healing assay, was performed to prove the enhanced effect of CoQ10 on skin regeneration when F2 was used in vitro. The cell scratch assay is performed to observe the wound healing and repair process, and thus this assay was utilized in keratinocytes and fibroblasts in our experiment. In both cell lines, the cells migrated towards the provisional space after scratching, which was monitored for 36 h. Each image of the keratinocyte (Figure 5a) and fibroblast (Figure 5b) was presented in Figure 5. The control group (the upper line) indicated the spontaneous migration rate of cells, demonstrating a relatively slow rate of migration and a relatively low wound healing effect compared to the F2-treated group (the lower line). In both cells, the F2-treated group indicated a rapid wound healing efficacy compared to the control group. In HaCaT cells, for example, F2 accelerated wound closure, resulting in a 98.95% of wound healing area following a 24 h exposure under microscopic observation (Figure S3). At the same time point, cells in the control group demonstrated 55.44% of wound healing area. Furthermore, this effect of F2 (72.31%) was also observed in the NIH3T3 cells compared to the control (48.61%). Collectively, the microemulsion containing CoQ10 accelerated wound healing in dermal cells, leading to skin regeneration.

## 4. Discussion

In the human body, the skin acts as a protective barrier against potential extrinsic factors [31]. In general, the skin has a regenerative function, leading to natural wound healing activity through complex physiological responses when the skin is injured and cut [32]. However, such a repair system is often insufficient for wound healing under normal conditions. Hence, the use of active ingredients is required to accelerate the regeneration rate and improve the physiological repair processes. However, active ingredients with wound healing activities are not suitable for topical applications due to the fact of their physicochemical properties such as a high molecular weight and inappropriate hydrophobicity (e.g., log *P*) [33,34]. For example, CoQ10 used in this study has a large molecular weight (863.3 g/mol), high lipophilicity (log *P* = 21), and poor solubility (e.g., 0.7 ng/mL at 37 °C). These properties are not suitable for topical administration, leading to low permeation, and low bioavailability. Therefore, in this study, the o/w microemulsion was chosen as a promising vehicle due to the fact of its increased solubility as well as thermodynamic stability [35,36]. Furthermore, the microemulsion demonstrated ease of manufacturing and scale-up after optimizing formulations. Previously, a self-emulsifying drug delivery system (SMEDDS) has been reported for oral administration of CoQ10. However, the SMEDDS still showed a low solubility of CoQ10 [37]. A nanoemulsion containing CoQ10 using Tween 80 was developed for alleviating the formation of wrinkles [38]. Another study reported that a CoQ10 microemulsion using glycosphingolipids demonstrated high CoQ10 solubility and improved bioavailability when orally administered, with CoQ10 used as a food ingredient and not a therapeutic agent [39]. Considering previous approaches, the emulsion system is still promising as a delivery vehicle for CoQ10 due to the fact of its low solubility. However, most formulations were not suitable for the topical delivery of CoQ10, resulting in the need to optimize new formulations to penetrate through the skin barrier and demonstrate skin regenerative efficacy after treatment. Therefore, we screened various oils, surfactants, and co-surfactants to select appropriate substances and their compositions for topical application.

Based on the pseudo-ternary phase diagrams, IPM as an oil, Cremophor EL^®^ as a surfactant and Transcutol^®^ HP as a co-surfactant were selected and their compositions and ratios were optimized after evaluating 864 formulations (data are not shown). Among the 24 formulations additionally selected, three kinds of formulations (F2, F10 and F18) were eventually selected, although they had relatively small amounts of surfactants and co-surfactant. Previous studies have reported that IPM as oil has been widely used for topical medications as well as cosmetic applications [40,41]. In this study, IPM experimentally demonstrated the highest CoQ10 solubility, approximately 4 fold higher than OA and Labrafil M1944, and is widely used in preparing microemulsions [42,43]. According to previous reports, Cremophor EL^®^, also known as Kolliphor EL (HLB value = 13.5), has often been investigated owing to its better emulsifying capacity than the Tween series [44]. Cremophor EL^®^ demonstrates enhanced permeability and bioavailability, although it is a nonionic surfactant [45]. Based on our data, Cremophor EL^®^ showed a higher solubility than Labrasol and Tween 80. As a co-surfactant, Transcutol^®^ HP, with an alcohol molecule, displayed a higher CoQ10 solubility than PG and PEG-400. A previous study reported that Transcutol^®^ is an effective enhancer for skin penetration and permeation [46] which correlated with our results. The selected formulations, including F2, F10, and F18, demonstrated thermodynamic stability and similar physicochemical properties prior to release and permeation tests. Selected formulations showed similar physiological properties as shown in Table 3. They were approximately 16 nm sized oil droplets with a slightly negative zeta potential. To select optimal composition of microemulsion, permeation experiments with semipermeable membrane as well murine skin were performed as shown in Figure 4. Based on in vitro data using a semipermeable membrane, their release, drug flus, and permeability of CoQ10 were analyzed using zero-order, first-order, Higuchi, and Krosemeyer–Peppass mathematical models (Supplementary Materials Table S1). Additional studies demonstrated that F2 with an S/CoS ratio of 2:1 demonstrated a higher permeation efficacy through the skin compared to F10 with an S/CoS ratio of 3:1 and F18 with an S/CoS ratio of 4:1. A marked increase in the amount of Transcutol^®^ HP could enhance the release as well as the skin permeation of CoQ10, confirmed in the in vitro release test and Franz cell diffusion test. The microemulsion with an S/CoS ratio of 2:1 demonstrated a higher release profile and permeation as Transcutol^®^ HP, as the co-surfactant, decreases the interfacial tension and increases the fluidity of the interface by reducing the bending stress [47]. Compared to F10 and F18, F2 had a relatively higher amount of CoS, leading to enhanced release of CoQ10 due to the reduction of S/CoS synergism. As the relative amount of the CoS increases, the efficiency of S/CoS to preserve the interface integrity during dilution is generally reduced, whereas the risk for destabilization and release rate increases [48].

Additionally, microemulsion improved the solubility of CoQ10 into the system and could be used as a skin permeation enhancer, presumably leading to the accumulation of CoQ10 in the skin. In particular, F2 selected showed a higher thermodynamic stability as well as improved release and permeation of CoQ10. In F2-treated cells, skin regeneration was additionally evaluated using in vitro cell experiments in which keratinocytes and fibroblasts were utilized, since they are the dominant cells in the wound closure mechanisms [49]. Compared to the control, F2 improved the skin regeneration efficacy in both cell lines due to the relative percent wound healing area (Figure 5 and Figure S3). Collectively, our findings demonstrated that F2 composed of IPM and an S/CoS ratio of 2:1 was an optimal microemulsion in the given experiment and demonstrated effective skin regeneration efficacy. As such, microemulsions have the ability of skin penetration by diffusion resulting from perturbing a structure of stratum corneum or by enhancing a drug solubility resulting from increasing the partition coefficient of drug between microemulsion and skin. When microemulsion was delivered via skin, it could show topical effect, resulting in different fate of drug. Based on our study, CoQ10 showed a topical delivery effect, presumably resulting in most CoQ10 was localized in skin layer.

## 5. Conclusions

Our findings demonstrated that the microemulsion was an effective carrier of CoQ10, with thermodynamic stability. The optimized CoQ10-loaded microemulsion demonstrated an improved release profile and enhanced skin accumulation after topical administration. In addition, the microemulsion indicated better wound healing efficacy in keratinocytes and fibroblasts, important cells for skin regeneration. In light of this, such microemulsions could be an attractive carrier for pharmaceutical and cosmetic applications due to the fact of their thermodynamic stability and excellent solubilizing property which could be applied for other pharmaceutical ingredients. They could be topically applied without toxicity and skin irritation. As such, the use of microemulsion as drug carrier have potential benefits over traditional formulations (e.g., cream, lotion, gel) from the scientific aspect due to the high solubilizing capacity and enhanced penetration efficacy, as well as from the industrial aspects due to the low preparation cost and ease of scale-up which could be more widely applied for topical and transdermal applications.

## Figures and Tables

**Table 1:** Solubility of CoQ10 in various oils, surfactant, and co-surfactant at room temperature (mean ± SD, *n* = 3).

Substance	Solubility (mg/mL)	Substance	Solubility (mg/mL)
Isopropyl myristate (IPM)	209.88 ± 7.42	Tween 80	21.88 ± 12.06
Oleic acid (OA)	87.36 ± 34.59	Cremophor EL^®^	33.10 ± 11.71
IPM:OA (3:1)	143.50 ± 18.97	Labarasol	26.53 ± 12.88
IPM:OA (2:1)	137.53 ± 14.17	PG	2.13 ± 2.13
Labrafil M1944	86.80 ± 12.98	Transcutol^®^ HP	9.35 ± 1.96
Cottonseed oil	107.12 ± 23.16	PEG-400	0.24 ± 0.21

**Table 2:** Compositions, drug loading, and thermodynamic stability test of formulations 1 to 24 o/w microemulsions.

Formulation	S/CoS ratio	Composition of ME *w*/*w* % *			Drug (%)	Dilution Test	Thermodynamic Stability Test			
		Oil	S/CoS	Water			Centrifugation	Heating-Cooling	Freeze-Thaw	Decision
F1	2:1	5.06	35.44	59.5	0.5	Clear	√	√	√	Pass
F2		5.06	35.44	59.5	1	Clear	√	√	√	Pass
F3		6.56	32.79	60.65	0.5	Clear	√	×	-	Failed
F4		6.56	32.79	60.65	1	Clear	√	×	-	Failed
F5		5.8	40.58	53.62	0.5	Clear	√	√	△	Failed
F6		5.8	40.58	53.62	1	Clear	√	√	△	Failed
F7		5	45	50	0.5	Clear	√	√	×	Failed
F8		5	45	50	1	Clear	√	√	×	Failed
F9	3:1	5.06	35.44	59.5	0.5	Clear	√	√	√	Pass
F10		5.06	35.44	59.5	1	Clear	√	√	√	Pass
F11		6.56	32.79	60.65	0.5	Clear	√	△	×	Failed
F12		6.56	32.79	60.65	1	Clear	√	△	×	Failed
F13		5.8	40.58	53.62	0.5	Clear	√	△	△	Failed
F14		5.8	40.58	53.62	1	Clear	√	△	△	Failed
F15		5	45	50	0.5	Clear	√	×	-	Failed
F16		5	45	50	1	Clear	√	×	-	Failed
F17	4:1	5.06	35.44	59.5	0.5	Clear	√	√	√	Pass
F18		5.06	35.44	59.5	1	Clear	√	√	√	Pass
F19		6.56	32.79	60.65	0.5	Clear	√	△	×	Failed
F20		6.56	32.79	60.65	1	Clear	√	△	×	Failed
F21		5.8	40.58	53.62	0.5	Turbid	-	-	-	Failed
F22		5.8	40.58	53.62	1	Turbid	-	-	-	Failed
F23		5	45	50	0.5	Turbid	-	-	-	Failed
F24		5	45	50	1	Turbid	-	-	-	Failed

* Oil; IPM, S/Cos; Cremophor EL^®^:Transcutol^®^ HP; √ is judged as “pass”; △ is judged as “poor flowability, poor turbidity; × is judged as “excess viscous behavior ” or “excess milky color (unstable agglomeration)”; Microemulsions were judged as “pass” when formulations was stable, whereas they were judged as “failed” when formulations was unstable after dilution test as well as thermodynamic tests.

**Table 3:** Physicochemical properties of selected formulations (Mean ± SD, *n* = 3).

Formulation	S/CoS Ratio	Composition of ME *w*/*w* % *			Drug (%)	Physicochemical Properties				
		Oil	S/CoS	Water		Droplet Size (nm)	PDI	Zeta Potential (mV)	Drug Content(%)	pH
F2	2:1	5.06	35.44	59.5	1	16.89 ± 0.050	0.040 ± 0.012	−13.1 ± 1.32	102.01 ± 0.128	7.12 ± 0.064
F10	3:1	5.06	35.44	59.5	1	16.72 ± 0.055	0.058 ± 0.007	−14.7 ± 1.23	101.7 ± 0.41	7.01 ± 0.021
F18	4:1	5.06	35.44	59.5	1	16.65 ± 0.172	0.080 ± 0.006	−11.8 ± 1.01	101.62 ± 0.4	7.00 ± 0.042

* Oil; IPM, S/Cos; Cremophor EL^®^:Trasscutol^®^ HP.

## Supplementary Materials

The following are available online at https://www.mdpi.com/1999-4923/12/4/332/s1, Figure S1. Release of CoQ10 using dialysis membrane. Figure S2. Cell proliferation in HaCaT (a) and NIH3T3 (b). Figure S3. Wound healing area % of skin cells after treatment of F2. Table S1. Release kinetics of CoQ10 from microemulsions.
