# Supplementary material for: Topical Delivery of Coenzyme Q10-Loaded Microemulsion for Skin Regeneration
Source: Pharmaceutics. 2020 Apr 7;12(4):332. doi: 10.3390/pharmaceutics12040332 (PMC7238272; doi:10.3390/pharmaceutics12040332)
Supplement: Supplementary file 1 [file pharmaceutics-12-00332-s001.pdf]

pharmaceutics

Supplementary Materials: Topical Delivery of Coenzyme Q10-Loaded Microemulsion for Skin Regeneration

Kyeong-A Ryu, Phil June Park, Seong-Bo Kim, Bum-Ho Bin, Dong-Jin Jang and Sung Tae Kim

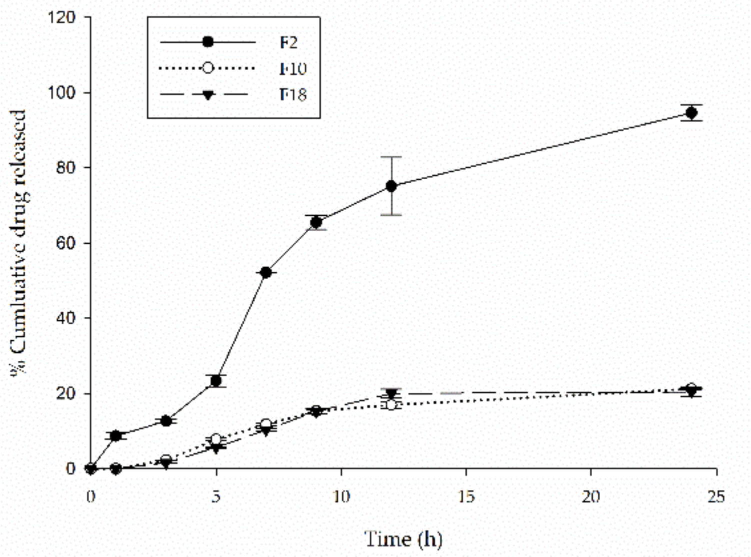

Figure S1. Release of CoQ10 using dialysis membrane. The release of CoQ10 from microemulsions (F2, F10 and F18) selected from 24 formulations was monitored for 1day. In vitro release was performed by a dialysis membrane (Slide-A-Lyzer™, Thermo Fisher Scientific, USA).

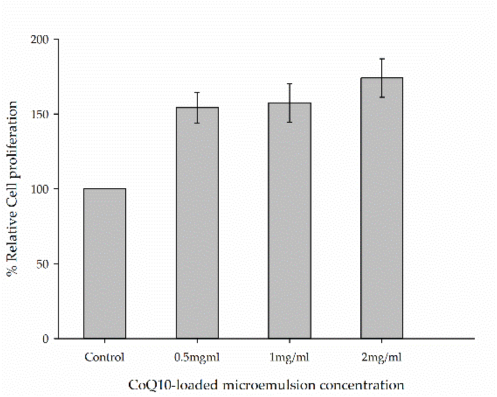

(**a**)

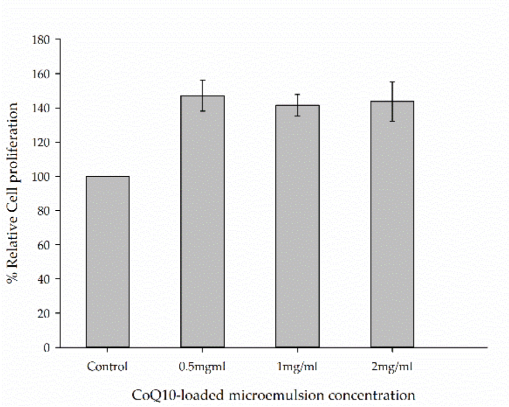

(**b**)

Figure S2. Cell proliferation in HaCaT (**a**) and NIH3T3 (**b**). Cell proliferation was performed when F2 was treated in both cell lines, which was measured by a cell counting kit-8 (CCK-8) at various concentration from 0.5 to 2 mg/mL for 1 day.

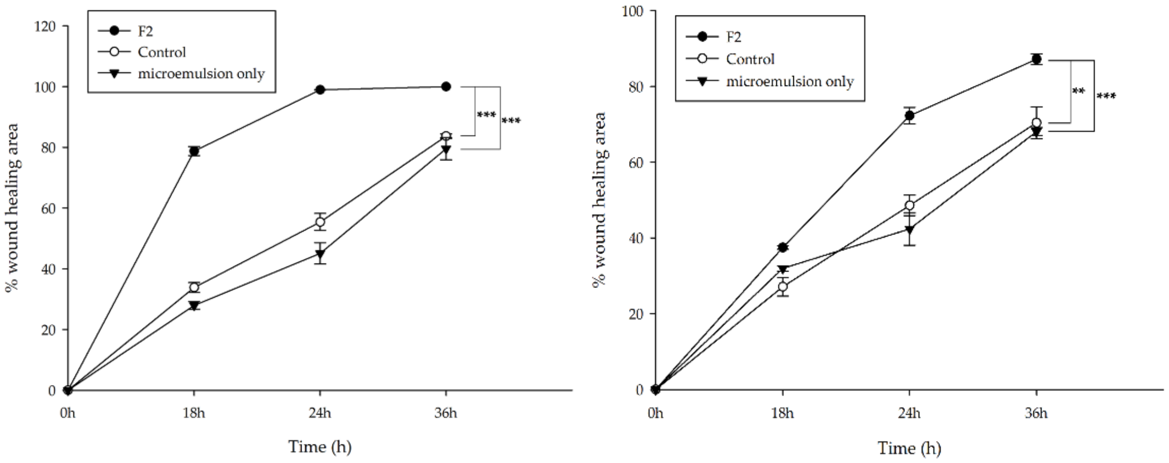

Figure S3. Wound healing area % of skin cells after treatment of F2. Each wound healing area % was calculated and compared based on scratched assay, which was monitored 36 hours after treatment of F2. Wound healing area in HaCaT (the left) and NIH3T3 (the right) was measured by calculating the wound area in each period and presented as a % of initial wound area. Data were presented as mean ± S.D. (n = 3); **, $p < 0.01$;***, and $p < 0.001$, respectively.

Table S1. Release kinetics of CoQ10 from microemulsions.

Formulation	Cumulative COQ10 Amount %	Kinetic of CoQ10 Release								Order of Release
		Zero-Order		First-Order		Higuchi		Krosemeyer-Peppas		
		R^2	K_0	R^2	K_1	R^2	K_H	R^2	n	
F2	102.88 ± 0.709 (24 h)	0.854	4.287	0.917	0.084	0.974	21.001	0.505	0.647	Higuchi
F10	84.66 ± 0.175 (24 h)	0.944	3.527	0.968	0.080	0.983	17.280	0.680	0.739	Higuchi
F18	74.58 ± 0.118 (24 h)	0.932	3.108	0.979	0.078	0.987	15.224	0.715	0.763	Higuchi
